# GABA_A _receptor γ2 subunit knockdown mice have enhanced anxiety-like behavior but unaltered hypnotic response to benzodiazepines

**DOI:** 10.1186/1471-2202-6-30

**Published:** 2005-04-25

**Authors:** Dev Chandra, Esa R Korpi, Celia P Miralles, Angel L De Blas, Gregg E Homanics

**Affiliations:** 1 Department of Anesthesiology, University of Pittsburgh School of Medicine, Pittsburgh, PA, USA; 2 Department of Pharmacology, Institute of Biomedicine, Biomedicum Helsinki, University of Helsinki, Helsinki, Finland; 3 Department of Physiology and Neurobiology, University of Connecticut, Storrs, CT, USA; 4 Department of Pharmacology, University of Pittsburgh School of Medicine, Pittsburgh, PA, USA

## Abstract

**Background:**

Gamma-aminobutyric acid type A receptors (GABA_A_-Rs) are the major inhibitory receptors in the mammalian brain and are modulated by a number of sedative/hypnotic drugs including benzodiazepines and anesthetics. The significance of specific GABA_A_-Rs subunits with respect to behavior and *in vivo *drug responses is incompletely understood. The γ2 subunit is highly expressed throughout the brain. Global γ2 knockout mice are insensitive to the hypnotic effects of diazepam and die perinatally. Heterozygous γ2 global knockout mice are viable and have increased anxiety-like behaviors. To further investigate the role of the γ2 subunit in behavior and whole animal drug action, we used gene targeting to create a novel mouse line with attenuated γ2 expression, i.e., γ2 knockdown mice.

**Results:**

Knockdown mice were created by inserting a neomycin resistance cassette into intron 8 of the γ2 gene. Knockdown mice, on average, showed a 65% reduction of γ2 subunit mRNA compared to controls; however γ2 gene expression was highly variable in these mice, ranging from 10–95% of normal. Immunohistochemical studies demonstrated that γ2 protein levels were also variably reduced. Pharmacological studies using autoradiography on frozen brain sections demonstrated that binding of the benzodiazepine site ligand Ro15-4513 was decreased in mutant mice compared to controls. Behaviorally, knockdown mice displayed enhanced anxiety-like behaviors on the elevated plus maze and forced novelty exploration tests. Surprisingly, mutant mice had an unaltered response to hypnotic doses of the benzodiazepine site ligands diazepam, midazolam and zolpidem as well as ethanol and pentobarbital. Lastly, we demonstrated that the γ2 knockdown mouse line can be used to create γ2 global knockout mice by crossing to a general deleter cre-expressing mouse line.

**Conclusion:**

We conclude that: 1) insertion of a neomycin resistance gene into intron 8 of the γ2 gene variably reduced the amount of γ2, and that 2) attenuated expression of γ2 increased anxiety-like behaviors but did not lead to differences in the hypnotic response to benzodiazepine site ligands. This suggests that reduced synaptic inhibition can lead to a phenotype of increased anxiety-like behavior. In contrast, normal drug effects can be maintained despite a dramatic reduction in GABA_A_-R targets.

## Background

GABA_A_-Rs are the major inhibitory receptors in the mammalian central nervous system and can be modulated by a number of sedative, hypnotic and anesthetic drugs [1]. GABA_A_-Rs are putatively pentameric complexes and are members of a 'superfamily' of related ligand-gated ion channels. There are a variety of subunit families that make up GABA_A_-Rs; a total of seventeen distinct subunits have been cloned, α1–6, β1–4, γ1–3, δ, ε, π, and θ. Much is known of the subunits that comprise GABA_A_-Rs, but the contribution of individual subunits to *in vivo *drug responses is only beginning to be understood, largely through the use of genetically engineered mice [2-4].

The γ2 subunit is highly expressed throughout developing and adult brain and spinal cord, is a component of almost 60% of all GABA_A_-Rs [5,6], and is required for synaptic clustering of GABA_A_-Rs [7]. *In vitro *electrophysiologic studies have demonstrated an obligatory role of the γ2 subunit for benzodiazepine responsiveness [8]. Gunther et al. created and studied mice that globally lacked all γ2 subunits of the GABA_A_-R [9]. Mice homozygous for the γ2 knockout died in the perinatal period with rare survivors reaching postnatal day 18. Knockouts appeared normal at birth, but developed sensorimotor abnormalities characterized by hyperactivity, impaired grasping and righting reflex, and abnormal gait. Pharmacologically, knockout of γ2 resulted in a severe deficit in benzodiazepine binding sites (~94% reduction compared to control) while GABA binding sites were only slightly reduced. Behaviorally, in the few mice that survived long enough to be tested, diazepam failed to induce sedation and loss of the righting reflex at doses that were fully effective in wild type mice [9]. γ2 heterozygous knockout mice were normally viable, had a ~50% reduction in γ2 protein levels, and displayed increased anxiety-like behaviors [10]. Drug responses of heterozygous γ2 global knockouts have not been reported, except for the high efficacy of low diazepam doses to induce anxiolysis [10].

We set out to make mice with markedly attenuated expression of the γ2 gene (i.e., γ2 knockdown mice) that would be viable and therefore useful for studies of basic behaviors and drug induced responses. We used gene targeting and embryonic stem cell technologies to create this mouse line by inserting a neomycin resistance gene (NEO) into intron 8 of the γ2 genomic locus. Insertion of NEO into intronic DNA has previously been used to create hypomorphic alleles of several genes [11-23]. In those studies, expression of the hypomorphic allele ranged from 5–25% of control; substantially lower than the 50% reduction typically observed in heterozygous knockouts. The γ2 knockdown mouse line described in the present report had a highly variable reduction in γ2 mRNA and protein levels. γ2 knockdown mice were normally viable, had increased anxiety-like behaviors, but did not differ in the hypnotic response to benzodiazepines. In addition, since exon 8 of the γ2 gene was flanked by loxP sites in this mouse line, these mice could also be converted into a global knockout mouse by crossing to a general deleter Cre-expressing transgenic mouse line.

## Results

### Production of gene targeted mice

**Figure 1:**
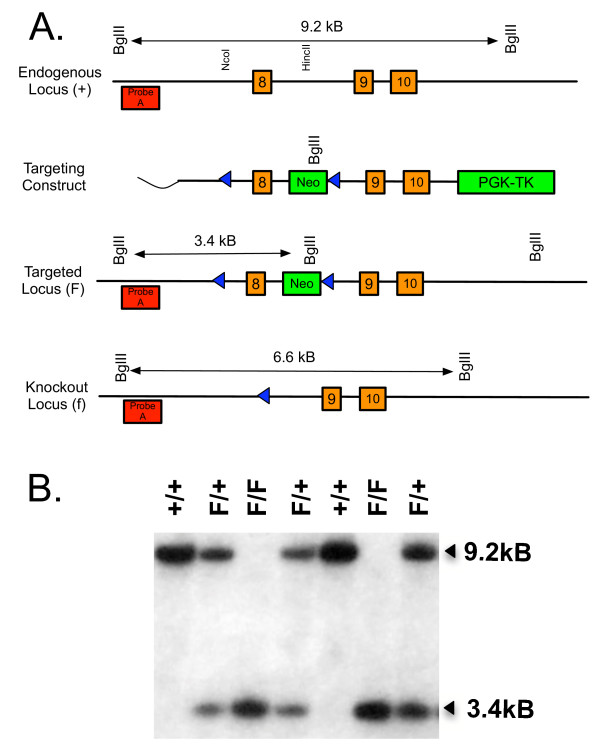
Gene targeting of the γ2 subunit of the GABA_A_-R and development of γ2 knockdown mice. (A) Targeting strategy used to produce γ2 knockdown mice. Relevant region of endogenous gene (+), targeting vector, correctly targeted knockdown γ2 (F), and cre-recombined knockout alleles (f) are shown. Relative locations of relevant restriction sites, exons (numbered orange boxes), loxP sites (blue triangles), plasmid backbone (wavy line), and positive (NEO) and negative (PGK-TK) selection cassettes are shown. (B) Southern Blot analysis of BglII digested tail DNAs. Probe A hybridizes to a 9.2 kb BglII fragment from the wild type endogenous allele and a 3.4 kb BglII fragment from the targeted allele.

Four out of 330 embryonic stem cell clones displayed the predicted restriction fragment length polymorphisms (data not shown) indicative of the targeting event presented in Fig 1A. Because the targeting event results in a γ2 locus in which exon 8 is flanked by loxP sites, we refer to this locus as being floxed (F) in contrast to the endogenous wild type allele (+). Two correctly targeted cell lines, 26L6 and 26C8 yielded germ-line competent chimeric males. Chimeras were mated to C57BL/6J females. Offspring that were +/F were interbred to produce +/+, +/F, and F/F mice. Mice were genotyped at weaning using Southern blot analysis as indicated in Fig 1B. In this analysis, Probe A hybridized to a 9.2 kB BglII fragment from the wild type γ2 allele and a 3.4 kB fragment from the targeted allele. Of 724 mice genotyped, 196 (27%) were +/+, 372 (51%) were +/F, and 156 (22%) were F/F. These values are in accord with the 1:2:1 genotype frequency as expected by Mendelian genetics. Thus, homozygous floxed mice were normally viable. They were also healthy and overtly indistinguishable from wild type mice.

### Northern blot analysis

**Figure 2:**
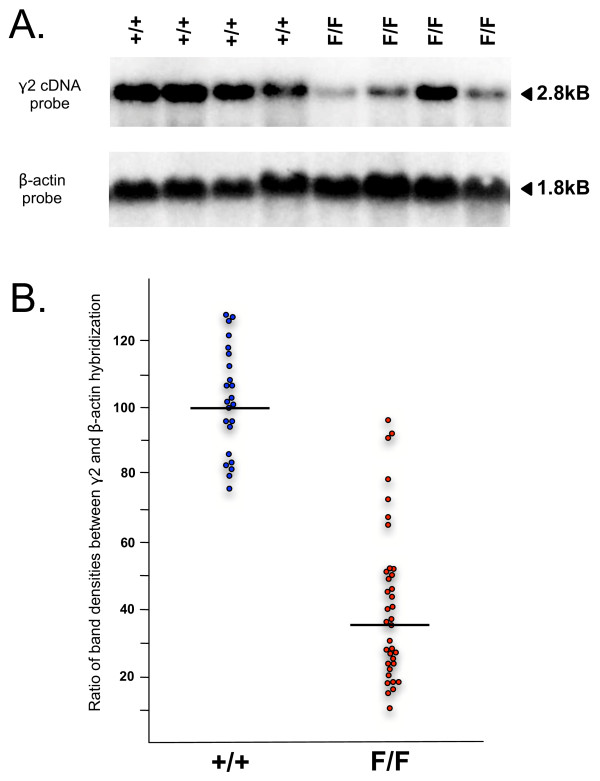
Northern blot analysis. (A) Sample northern blot analysis of wild type (+/+) and homozygous knockdown (F/F) adult whole brain total RNA hybrizided with a γ2 cDNA or a human β-actin probe as a loading control. (B) Densitometric analysis of northern blots. The ratio of band density between γ2 and β-actin hybridization for wild type (n = 23) and homozygous knockdown (n = 36) mice was plotted. On each individual blot, the ratio of wild type band densities was averaged and normalized to 100. All samples on each blot were then compared to the average of wild type band densities on that blot. A total of 4 different blots were included in this analysis. The horizontal bar in each column represents the mean for that genotype.

Semi-quantitative northern blots were used to compare the amount of γ2 mRNA in wild type and F/F animals. Hybridization of adult brain RNA with a γ2 cDNA probe yielded an intense 2.8 kB band in wild type mice. In contrast, RNA from F/F mice hybridized less intensely in most mice, however the attenuation within this group was highly variable. A sample northern blot is shown in Figure 2A. We semi-quantitatively determined γ2 mRNA amounts by comparing γ2 band densities between wild type (n = 23) and F/F (n = 36) mice following normalization with β-actin. To normalize results over four different blots, the ratio of wild type band densities on each blot was averaged and normalized to 100. All samples on each blot were then compared to the average of wild type band densities. Values are shown in Figure 2B. Mean wild type ratio was 100 whereas mean ratio in F/F samples was 35 with high variability including several mice with values that overlapped with wild type values. Thus, γ2 mRNA levels in knockdown mice were dramatically attenuated on average, but the levels varied significantly between mice.

### Immunohistochemistry

**Figure 3:**
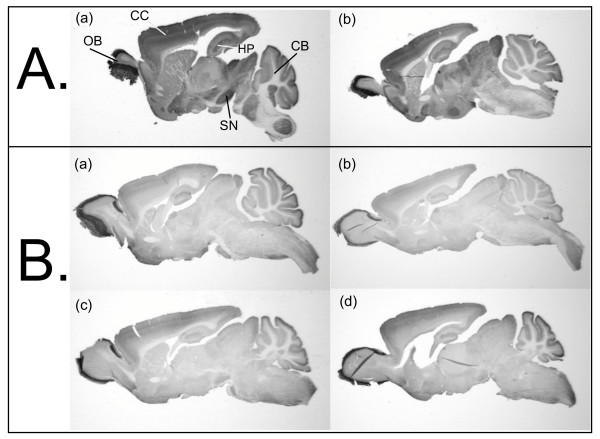
Immunohistochemical distribution of γ2 subunit of the GABA_A_-R in sections from individual (A) wild type and (B) homozygous knockdown mice. Note the high level of abundance of γ2 immunoreactivity in olfactory bulb (OB), cerebral cortex (CC), hippocampus (HP), substantia nigra (SN), and cerebellum (CB) of wild type samples. The amount of γ2 is variably reduced in many brain regions of the knockdown samples.

Immunohistochemical staining of sagittal sections probed with a γ2-specific antibody was used to assess γ2 protein amount and distribution in the brain. A sampling of wild type (Fig 3A) and knockdown (Fig 3B) brain sections are displayed. In three of the four knockdown mouse sections shown, there was a marked overall reduction of γ2 immunoreactivity in all areas of the brain except the outer layer of the olfactory bulb. However, staining intensity was variable between knockdown mice, with one having near wild type intensity (Fig. 3B, d). A total of 10 F/F mice were studied. Seven out of 10 had a marked decrease in γ2 staining compared to wild type controls, and three out of 10 had near wild-type or intermediate levels of staining. Adjacent sections from these same mice showed no change in α1 or β2/3 immunoreactivity (CPM and ALD, unpublished observations).

### Pharmacological characterization

Autoradiography on brain cryostat sections with various ligands binding to selective sites of the GABA_A_-R revealed clear differences in the homozygous γ2 knockdowns as compared to heterozygous and wild type mice (Table 1). Total flumazenil-sensitive [^3^H]Ro15-4513 binding to benzodiazepine sites was reduced in the knockdown in most brain regions. The reduced binding in the 5 mice analyzed ranged on the average from 25 to 57%. In several regions, the heterozygous mice also showed significant reduction of the binding, but clearly less than the homozygous knockdown samples. A small proportion of the reduction in the total binding was apparently due to reduction in diazepam-insensitive α4 and α6 subunit-containing receptors, this component being mostly affected in the cerebellar granule cell layer.

GABA-sensitive [^3^H]muscimol binding to GABA agonist sites was not altered in any brain region (Table 1).

Picrotoxinin-sensitive [^35^S]TBPS binding to the GABA_A_-R channels was little affected between the mouse lines: basal binding was increased in the hippocampus and decreased in the cerebellar granule cell layer of the homozygous γ2 knockdowns (Table 1). The GABA-insensitive [^35^S]TBPS binding was increased all over the brain, but this component still made up less than 10% of the basal binding, except for the cerebellar granule cell layer. There, the GABA-insensitive component was 20% as compared to 10% in the heterozygous and wild type animals.

### Behavioral characterization

**Figure 4:**
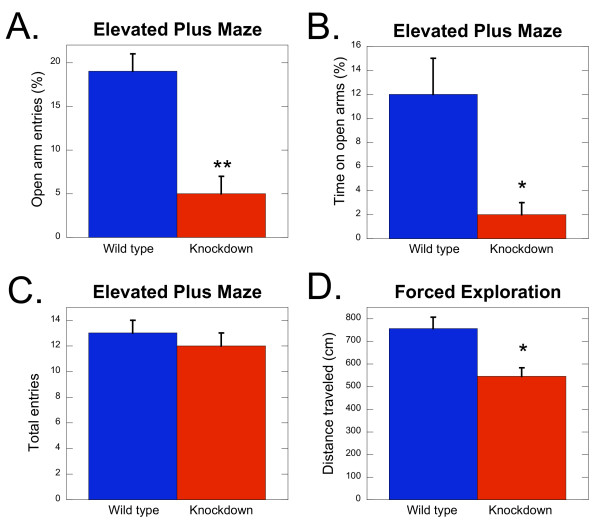
Behavioral characterization of knockdown mice. (A-C) Elevated plus maze. Knockdown mice (A) entered open arms less often and (B) for less time, however, (C) total entries into arms did not differ from wild type mice. (D) Knockdown mice were also less active in the forced exploration test. The bars are means ± SEM. *p < .01, **p < .001.

We examined the behavioral phenotype of the knockdown mice using the elevated plus maze and forced exploration in a novel open field. The elevated plus maze is widely used to assess anxiety-like behavior in response to the aversive stimulus of an elevated exposed space. On this test apparatus, knockdown mice had a 74% reduction in open arm entries (Fig. 4A) and an 83% reduction in time on open arms (Fig. 4B) compared to controls suggesting an increase in anxiety-like behavior. In contrast, knockdown mice had the same number of total arm entries as wild types (Fig. 4C) indicating that an alteration in locomotion does not account for the differences in anxiety-like behavioral measures on this assay.

Knockdown mice were also tested for behavioral response to an aversive, brightly lit open field test apparatus using the forced novelty exploration test. Knockdown mice had a 28% reduction in locomotor activity compared to wild type mice when placed into this test arena (Fig. 4D). This reduction in exploratory behavior in the knockdown animals is also indicative of an increase in anxiety-like behavior.

**Figure 5:**
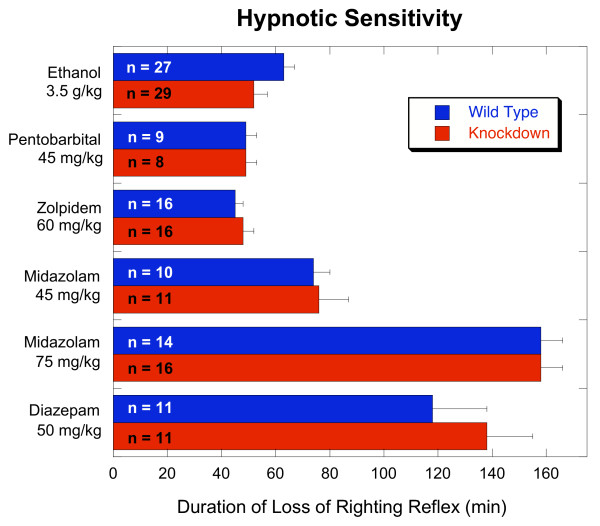
Hypnotic sensitivity. No significant differences in the hypnotic effects of ethanol, pentobarbital, zolpidem, midazolam, or diazepam were observed. The bars are means ± SEM.

Knockdown of the γ2 subunit did not alter hypnotic responses to benzodiazepine site ligands or other sedative/hypnotic drugs tested. We measured the loss of righting reflex to assess the acute sensitivity to sedative/hypnotic drugs; no significant differences were found in response to injection of diazepam, midazolam, zolpidem, ethanol, or pentobarbital (Fig. 5).

### Production of γ2 global knockout mice

The gene targeting strategy that we used to produce the γ2 knockdown mice also resulted in the insertion of loxP sites that flank exon 8 of the γ2 gene (see Fig. 1A). To determine if these loxP sites were functional *in vivo*, heterozygous floxed γ2 mice were mated to a general deleter cre-expressing transgenic mouse line (JAX Laboratory, stock #003376) where the cre transgene was driven by a ubiquitously expressed human beta actin promoter [24]. Restriction fragment polymorphism analysis by Southern blotting confirmed the deletion of genomic DNA between loxP sites. As predicted from Fig. 1A, Probe A hybridized to a 6.6 kb BglII fragment from the recombined knockout allele (data not shown).

To determine if cre-mediated recombination and deletion of exon 8 resulted in a null allele, mice hemizygous for the cre transgene and heterozygous for the recombined γ2 allele (+/f) were mated to mice heterozygous for the floxed, non-recombined γ2 allele (+/F). 10.2% (14 of 137) of offspring from this mating strategy were homozygous for the recombined locus (f/f). This ratio is in agreement with the 12.5% predicted from Mendalian genetics. 13 of the 14 f/f mice died within three days of birth. One f/f mouse survived until postnatal day 21. This neonatal lethality of γ2 f/f mice is consistent with those found in the global γ2 knockouts of Günther et al. [9] which also had a deletion of exon 8. Therefore, we conclude that cre-mediated recombination of the loxP sites and deletion of exon 8 inactivates the γ2 gene.

## Discussion

Genetic dissection of the GABA_A_-R system is yielding remarkable insights into GABA_A_-R biology and the mechanisms of action of various drugs. Here we report the establishment of a new GABA_A_-R mutant mouse model, one with attenuated expression of the γ2 subunit that averages ~35% of normal levels. This novel γ2 knockdown model has several unique features that make it useful. First, compared to global knockouts which die as neonates [9], knockdown mice have normal viability. Secondly, the graded levels of expression of γ2 (range 10–95% of normal) in the mutant mice could be useful in studies correlating phenotype with the amount of γ2 protein present. Finally, global knockout mice can be created from these mice by crossing to a Cre recombinase expressing global deleter mouse line. Therefore, the mice reported here represent a useful addition to the rapidly expanding arsenal of mice with genetic alterations in the γ2 subunit of the GABA_A_-R. In addition to homozygous and heterozygous global γ2 knockouts [9], conditional γ2 knockout mice [25], knockin mice with a point mutation in γ2 that eliminates zolpidem and inverse agonist β-carboline sensitivity [26,27], global knockout of the long splice variant of γ2 [28], and transgenic mice that express either the long or short splice variants of the γ2 subunit [29] have been described.

An important unexpected finding from this study was the extremely variable degree to which the genetic alteration of the γ2 locus changed the amount of γ2 product produced. This γ2 knockdown model was produced by insertion of NEO into intronic DNA. This strategy has been used previously to create hypomorphic alleles [11-23]. Creation of hypomorphic alleles has been useful in determining function of the gene that has been targeted, especially in cases where complete disruption of the gene is lethal [12,14-16]. Intronic insertion of NEO creates hypomorphic alleles either through cryptic splicing signals in the NEO gene that lead to a frameshift or alterations in splicing, or by down regulating gene expression through an unknown mechanism [14-16]. In the majority of these studies, the authors' reported a 5–25% level of expression from the targeted gene. However, in none of these previously described hypomorphic alleles was extensive variability reported. The γ2 targeted homozygous mice in this study clearly display a wide range of γ2 mRNA and protein levels. Semi-quantitative northern blot analysis revealed that 4 out of 5 homozygous knockdown mice showed, on average, a 70% reduction of γ2 mRNA levels, whereas 1 out of 5 mice had γ2 mRNA at a level that was similar to wild type. Immunohistochemical data were consistent with northern blot data. The reason for this highly variable expression within homozygous knockdown mice has yet to be determined. The mixed background of Strain 129S1/X1 and C57BL6/J may not be the cause given that other studies have not observed substantial variability in expression using similar background strains [11,18,20]. Gender as a cause of variability also is not likely given that a set of three male knockdown siblings exhibited the full range of γ2 levels; one being near wild type levels, another being at an intermediate level, and the third exhibiting severely attenuated expression. Further study to determine the cause of this variability needs to be undertaken. Nevertheless, investigators who desire to create a hypomorphic allele by inserting NEO into intronic DNA should take into consideration the variability of gene expression observed in this study. The extent of variability may be locus dependent.

Knockdown of γ2 subunit containing GABA_A_-Rs resulted in behavioral changes that are indicative of increased anxiety-like behavior. These mutant knockdown mice showed reduced exploratory behavior for a novel open field apparatus and the open arms of the elevated plus maze. Similar changes have been observed in γ2 global heterozygous knockout mice [10]. Together, these results strengthen the possibility that GABA_A_-R dysfunction may be an underlying cause of anxiety disorders in humans. These mutant mouse models may be useful for dissecting the etiology of this pervasive condition and for developing effective therapeutic interventions.

As expected, brains from γ2 knockdown mice demonstrated significantly decreased binding of the benzodiazepine site ligand, Ro15-4513. This was expected since benzodiazepine binding sites are located at the interface of select alpha and γ2 subunits of the GABA_A_-R [30]. The reduction observed in Ro15-4513 binding agree with those from global γ2 knockout mice [9] in that deficiency of the γ2 subunit abolishes the binding of the benzodiazepine-site ligands. The global heterozygous γ2 knockout mice have a reduction in total [^3^H]Ro15-4513 binding in most brain regions [31], and the present data on the γ2 knockdown show a very similar pattern of reduced binding. However, the 5 knockdown brains that we examined had actually a greater reduction in benzodiazepine binding than the γ2 heterozygous global knockouts published earlier [31]. Similar to the γ2 heterozygous knockouts, [^3^H]muscimol binding was not affected, indicating that the reduction of the γ2 subunit levels is not compensated by increased δ subunit that is largely responsible for the [^3^H]muscimol binding signal in brain sections [32]. The basal binding of the ion channel ligand [^35^S]TBPS is usually reduced and its sensitivity to GABA increased by addition of γ2 subunits [31]. Both in the γ2 heterozygous global knockout and in our homozygous knockdown, TBPS binding was increased in some brain regions, and, more consistently, there emerged a "GABA-insensitive" receptor population with widespread distribution in the brain. This indicates production of GABA_A_-Rs with an αβ configuration. These receptors bind strongly the agonist and have reduced channel conductance [33], perhaps reflecting only partial agonist efficacy of GABA. This would explain the reduced allosteric efficacy of GABA in abolishing TBPS binding. In addition, these receptors have most likely non-synaptic, non-clustered localization as they lack the γ2 subunit [10,33]. Therefore, the partially γ2 depleted mice may have brain region-selective alterations in synaptic and extrasynaptic receptors, and the reduced synaptic GABA_A_-R function might correlate with the anxious phenotype. However, surprisingly, the high-dose hypnotic effects of the benzodiazepine site ligands diazepam, zolpidem and midazolam were unchanged in the knockdown mice.

Several hypotheses can be generated to explain the discrepancy between decreased benzodiazepine ligand binding and unchanged hypnotic effects. One possibility is that only a threshold level of γ2 containing GABA_A_-Rs are required for hypnotic responses to benzodiazepines. Mice that completely lack γ2 had only 6% of benzodiazepine binding compared to wild type mice and were insensitive to the hypnotic effects of diazepam [9]. Likewise, mice with a point mutation in γ2 that eliminated response to zolpidem eliminated the effects of this benzodiazepine site ligand [26]. It seems likely that the 20–40% of benzodiazepine sensitive receptors that remain in the knockdown mice are enough to mediate the hypnotic effect of benzodiazepine site ligands. This is supported by positron emission tomography studies in humans, in which only a small proportion of the benzodiazepine sites need to be occupied by lorazepam and zolpidem to induce clinical effects such as sedation [34,35]. There may thus be spare receptors that can be measured by biochemical techniques but are not needed for whole animal pharmacological effects. Another hypothesis is that knockdown of γ2 does not result in a proportional reduction in all γ2 containing GABA_A_-Rs. Recent studies conducted in knockin mice with a point mutation at the benzodiazepine binding site have attributed α1 and α2 containing GABA_A_-Rs to the mediation of benzodiazepine-induced sedation and anxiolysis, respectively [4,36-38]. Perhaps γ2 knockdown mice have disproportionate decreases in non-α1 containing receptors, e.g., α2 or α3 containing receptor isoforms. It may be possible that in our γ2 knockdown mice, the number of α2βxγ2 GABA_A_-Rs is greatly reduced while the number of α1βxγ2 receptors is not, which remains to be studied. This could be tested by immunohistochemical methods using antibodies against α2 and α3 or by testing for changes in zolpidem insensitive binding. Changes in subunit trafficking may also play a role. Essrich et al. [7] established strong evidence that GABA_A_-Rs are clustered at the synapse through indirect interactions between γ2 and gephyrin. Enough clustering may remain in the knockdown mice such that drug response does not change. Alternatively, synaptic clustering of GABA_A_-Rs may not be important for behavioral responses to benzodiazepines, and it is possible that benzodiazepines potentiate the extrasynaptic GABA_A_-R responses determined by electrophysiology in brain slices [39]. It is also possible that the high variability in γ2 levels in the brain of knockdown mice masked any change in behavioral response to these drugs. In hindsight, it would have been useful to quantify γ2 levels in the same mice that were used for the behavioral sensitivity studies. It would be interesting to see if sensitivity correlated with γ2 levels on an individual animal basis. This type of approach could be exploited in future studies of γ2 receptor function. Lastly, decreases in benzodiazepine binding may not directly translate into changes in *in vivo *insensitivity. For example, in α1 null mice, changes in benzodiazepine binding did not change whole animal sensitivity to midazolam but increased sensitivity to diazepam [40,41]. In contrast, in mice that selectively lack the long splice variant of γ2, affinity for benzodiazepine site ligands is increased and behavioral response to the sedative effects of midazolam and zolpidem is also increased [42]. Similarly, in mice lacking the β3 subunit, decreased benzodiazepine binding and decreased whole animal midazolam sensitivity were observed [43]. Further study is required to examine these possibilities.

## Conclusion

We have produced a novel genetically engineered mouse line that exhibits a reduction in γ2 mRNA levels that is highly variable between mice (range 10–95% of control) but averages ~35% of control levels. These γ2 knockdown mice are viable and have enhanced anxiety-like behavioral abnormalities. Surprising, these mice are normally sensitive to the hypnotic effects of benzodiazepine site ligands. Lastly, this novel genetically engineered mouse line can also be easily converted to a γ2 global knockout mouse line by simply crossing to a cre-expressing global deleter transgenic mouse line.

## Methods

### Generation of genetically altered mice

A 13.6 kB BglII Strain 129/SvJ mouse genomic DNA fragment containing Exons 8–10 of the GABA_A_-R γ2 gene was subcloned from a P1 phage clone (Genome Systems, St. Louis, MO). An oligonucleotide containing a loxP site and an EcoRV site was inserted into a NcoI site 647 bp 5' to Exon 8. A blunted fragment containing a NEO resistance gene with PGK-1 promoter and polyadenylation signals (obtained from pPNT [44]) fused with a loxP site was inserted into a HincII site 855 bp 3' of Exon 8. The NEO gene was inserted in the opposite orientation to γ2 transcription. A PGK driven thymidine kinase expression cassette (obtained from pPNT [44]) was then cloned 3' to the γ2 genomic DNA. The targeting construct was linearized with SalI and electroporated into R1 embryonic stem cells [45] following previously described procedures [46]. G418 (270 μg/ml; Life Technologies, Gaithersburg, MD) and gancyclovir (2 μM; Sigma) resistant cells were screened for gene targeting by Southern blot analysis of BglII digested genomic DNA. Fragments were hybridized with a 5' probe that was external to the targeting construct (see Fig. 1). Proper targeting of the γ2 locus was confirmed by Southern blot analysis of EcoRV and SstI digested DNA. Additional Southern blot analysis with probes to the neomycin cassette or internal to the 3' arm of the targeting vector (with 1–3 restriction enzymes) were also conducted (results not shown). All results were consistent with a correctly targeted γ2 locus.

Two correctly targeted embryonic stem cell clones (26C8 and 26L6) were microinjected into C57BL/6J blastocysts to produce chimeric mice. Male chimeras were mated to C57BL/6J females. Agouti offspring heterozygous for the targeted allele (F/+) were interbred to produce mice that were wild type (+/+), heterozygous, or homozygous mutants (F/F). The mice used in studies reported here were derived from the 26L6 ES cell clone. All mice were housed under conditions of lights on at 07:00 and lights off at 19:00.

### Northern blot analysis

Ten to fourteen week old mice were sacrificed by cervical dislocation. RNA was isolated from whole brain using Trizol reagent (Invitrogen). 20 μg of total RNA was electrophoresed in a 1.9% formaldehyde/1% agarose gel and blotted onto Hybond-N (Amersham Pharmacia). Blots were first probed with a ^32^P-labeled γ2 cDNA probe then re-hybrizided with a control human β-actin probe (Clontech). Autoradiographs were digitally photographed and band densities were measured using Kodak 1D Image Analysis Software. Ratio between density of γ2 hybridization versus actin hybridization was recorded.

### Immunocytochemistry

The procedure has been described elsewhere [47]. Briefly, wild type and homozygous mutants were deeply anesthetized with Avertin and transcardially perfused with Dextran-Phosphate Buffer (PB) then fixative consisting of 0.01M periodate/0.075M lysine/4% paraformaldehyde in 0.1M PB (pH 7.4). The brains were frozen and sliced in parasaggital sections (25 μm) with a freezing microtome. Slices were incubated overnight at 4°C with affinity-purified rabbit anti-γ2 [48,49] in 0.1M PB (pH 7.4) with 0.3% Triton X-100, at a concentration of (1:100). The sections were processed using an avidin-biotin-peroxidase system (Vectastain Elite; Novocastra, Burlingame, CA). Peroxidase reaction was carried out with 3-3' diaminobenzidine tetrahydrochloride in the presence of cobalt chloride and nickel ammonium sulfate as chromogens and hydrogen peroxide as oxidant. Controls were performed either by omitting the primary antibody or by incubating the primary antibody with the corresponding peptide [50].

### Pharmacology

Eight-week-old mice were sacrificed by decapitation and whole brains were rapidly dissected out and frozen on dry ice. For autoradiography, 14-μm horizontal serial cryostat sections were cut from 5 mouse brains of each genotype, thaw-mounted onto gelatin-coated object glasses, and stored frozen under desiccant at -20°C. All experiments were carried out in parallel fashion with respect to mouse lines, eliminating any day-to-day variation between the assays. The autoradiographic procedures for regional localization of [^3^H]Ro 15-4513, [^3^H]muscimol, and [^35^S]TBPS binding were as described in [51]. Briefly, sections were preincubated in an ice-water bath for 15 min in 50 mM Tris-HCl (pH 7.4) supplemented with 120 mM NaCl in the [^3^H]Ro 15-4513 and [^35^S]TBPS autoradiographic assays, and in 0.31 M Tris-citrate (pH 7.1) in the [^3^H]muscimol assay. All radioligands were purchased from Perkin Elmer Life Sciences, Inc. (Boston, MA, USA).

The final incubation in respective preincubation buffer was performed with 6 nM [^35^S]TBPS at room temperature for 90 min, assays with 10 nM [^3^H]muscimol at 0–4°C for 30 min, and assays with 10 nM [^3^H]Ro 15-4513 in the presence and absence of 100 μM diazepam (Orion Pharma, Espoo, Finland) at 0–4°C for 60 min. After incubation, sections were washed 3 × 15 s or 2 × 30 s in an ice-cold incubation buffer in [^35^S]TBPS and [^3^H]Ro 15-4513 or in [^3^H]muscimol assay, respectively. Sections were then dipped into distilled water, air-dried under a fan at room temperature, and exposed with plastic [^3^H]- or [^14^C]-methacrylate standards to Kodak Biomax MR films for 1 to 8 weeks. Nonspecific binding was determined with 10 μM flumazenil (Hoffmann-La Roche, Basel, Switzerland), 100 μM picrotoxinin (Sigma) and 100 μM GABA (Sigma) in [^3^H]Ro 15-4513, [^35^S]TBPS and [^3^H]muscimol assays, respectively. Binding densities in the relevant brain areas were quantitated with MCID M5-imaging software (Imaging Research Inc., Ontario, Canada) and converted to nCi/mg (for ^3^H) or nCi/g (for ^35^S) radioactivity values on the basis of the simultaneously exposed standards.

The concentrations of [^3^H]muscimol (10 nM) and [^3^H]Ro 15-4513 (10 nM) were greater than or equal to the dissociation constants for a range of recombinant and native GABA_A _receptors [8,52-54]. Therefore, the autoradiographic images should represent the density rather than affinity of binding sites.

### Hypnotic sensitivity

Drug induced hypnosis was assessed by measuring the duration of the loss of righting reflex in response to various sedative/hypnotic drugs. Diazepam (25 mg/kg; Sigma), midazolam (45 mg/kg and 75 mg/kg; ESI Lederle, Philadelphia, PA), pentobarbital (45 mg/kg; Abbott, Chicago, IL), zolpidem (60 mg/kg; Searle, Malvern, PA) or ethanol (3.5 mg/kg; Pharmco, Brookfield, CT) were administered intraperitoneally to wild type and homozygous mutants. Drugs were diluted in saline such that the injection volume was 20 μL per gram body weight. After losing the righting reflex, mice were placed in a plastic V-shaped trough and the time was recorded. When the mouse was able to right itself three times in 30 s, the measure of hypnotic effect was over. Body temperature was maintained with aid of a heat lamp. Assays were performed in a blinded manner with respect to genotype. Effect of genotype on the duration of the loss of righting reflex was compared using Student's *t*-test.

### Elevated plus-maze test

Basal anxiety-like behavior was tested using the elevated plus-maze. All mice were between 7 and 9 weeks of age and were tested between 09:00 and 11:00. Each mouse was placed on the central platform of the maze, facing an open arm and allowed to freely explore the maze for 5 min under ambient room light. Open-arm and closed-arm entries and the cumulative time spent on the open and closed arms was recorded. A mouse was considered to be on the central platform or on an arm when all four paws were within its perimeter. The percent open-arm entries, total number of entries, and percent time in open-arms was determined. Data were analyzed using one-way ANOVA.

### Forced exploration

Basal motor activity of mice was determined using the forced exploration test. 8–10 week old mice were placed into a walled arena (43.2 cm × 43.2 cm × 30.5 cm) for 5 min. Distance traveled (cm) was measured automatically using an Activity Monitor (Med Associates, St. Albans, VT). All tests were performed between 12:00 and 15:00. Effect of genotype on basal motor activity was compared using Student's *t*-test.

## Authors' contributions

CPM and ALD carried out the immunohistochemical studies. ERK carried out the ligand autoradiography studies. All other experiments were performed by DC and GEH. All authors contributed to composing and editing the manuscript and have read and approved the final version.

## Figures and Tables

**Table 1:** Autoradiographic analysis of GABA_A _receptor binding sites in horizontal sections of wild type and mutant mice.

**BRAIN REGION**							
**Ligand/condition**	**Ctx**	**Th**	**Hi**	**CPu**	**IC**	**Gr**	**Mol**

**[^3^H]Ro15-4513, basal**							
+/+ mice	102 ± 3	53 ± 3	102 ± 1	52 ± 4	63 ± 7	93 ± 4	84 ± 4
+/F mice	97 ± 2	42 ± 2^c^	91 ± 3^c^	44 ± 3^b^	52 ± 7	74 ± 7^b^	72 ± 7^a^
F/F mice	77 ± 6^c^	23 ± 2^c^	68 ± 5^c^	30 ± 3^c^	38 ± 13^b^	59 ± 7^a^	62 ± 6^c^
							
**[^3^H]Ro15-4513 + Diazepam 100 μM**							
+/+ mice	4.4 ± 0.3	4.0 ± 0.3	7.0 ± 0.4	3.7 ± 0.3	3.3 ± 0.2	56 ± 5	5.4 ± 1.0
+/F mice	3.8 ± 0.3^a^	3.5 ± 0.4	5.7 ± 0.5^c^	3.3 ± 0.3	3.3 ± 0.3	41 ± 5^c^	4.7 ± 0.9
F/F mice	3.1 ± 0.2^c^	2.8 ± 0.2^c^	4.3 ± 0.3^c^	2.8 ± 0.2^c^	2.9 ± 0.1^a^	13 ± 4^c^	3.7 ± 1.5
							
**[^3^H]Muscimol, basal**							
+/+ mice	16 ± 2	20 ± 4	9.3 ± 1.3	11 ± 2	4.2 ± 0.6	94 ± 7	8.7 ± 2.3
+/F mice	18 ± 2	23 ± 3	10.0 ± 2.1	11 ± 2	3.9 ± 0.8	101 ± 8	8.6 ± 1.0
F/F mice	15 ± 3	23 ± 7	9.7 ± 3.0	11 ± 4	3.8 ± 0.6	89 ± 16	8.1 ± 3.8
							
**[^35^S]TBPS, basal**							
+/+ mice	210 ± 26	583 ± 143	211 ± 30	257 ± 55	914 ± 41	641 ± 114	80 ± 25
+/F mice	271 ± 47	527 ± 110	248 ± 34	277 ± 31	866 ± 51	722 ± 140	73 ± 16
F/F mice	277 ± 57	460 ± 145	299 ± 43^b^	292 ± 50	829 ± 98	425 ± 32^b^	100 ± 30
							
**[^35^S]TBPS + GABA 1 mM**							
+/+ mice	3.0 ± 2.0	14 ± 9	1.7 ± 1.8	0.4 ± 2.6	4.6 ± 2.2	67 ± 6	1.7 ± 1.8
+/F mice	7.0 ± 1.3^a^	29 ± 13	5.9 ± 1.6^b^	4.9 ± 0.4^a^	18 ± 5a	75 ± 12	0.7 ± 2.4
F/F mice	16 ± 2^c^	42 ± 16^a^	13 ± 2.0^c^	18 ± 3^c^	76 ± 12^c^	86 ± 13^a^	5.0 ± 2.2

The data are means ± standard deviations for 5 mice in each genotype, and are expressed as nCi/mg for ^3^H-ligands and as nCi/g for ^35^S-ligand. Significance of the difference from wild type (Tukey-Kramer test after ANOVA): ^a^P < 0.05, ^b^P < 0.01, ^c^P < 0.001. Ctx, cortex; Th, thalamus; Hi, hippocampus; CPu, caudate putamen; IC, inferior colliculus; Gr, cerebellar granule cell layer; Mol, cerebellar molecular layer.
